# Macrophage-Conditioned Media Promotes Adipocyte Cancer Association, Which in Turn Stimulates Breast Cancer Proliferation and Migration

**DOI:** 10.3390/biom12121757

**Published:** 2022-11-26

**Authors:** Karin A. Vallega, Dale B. Bosco, Yi Ren, Qing-Xiang Amy Sang

**Affiliations:** 1 Department of Chemistry and Biochemistry, Florida State University, 95 Chieftan Way, Tallahassee, FL 32306-4390, USA; 2 Institute of Molecular Biophysics, Florida State University, 95 Chieftan Way, Tallahassee, FL 32306-4390, USA; 3 Department of Biomedical Sciences, Florida State University College of Medicine, 1115 West Call Street, Tallahassee, FL 32306-4300, USA

**Keywords:** 3D co-culture model, human breast cancer, macrophages, adipocyte, inflammation, paracrine interactions, obesity

## Abstract

Background: Breast cancer is the most common cancer in women and the leading cause of female cancer deaths worldwide. Obesity causes chronic inflammation and is a risk factor for post-menopausal breast cancer and poor prognosis. Obesity triggers increased infiltration of macrophages into adipose tissue, yet little research has focused on the effects of macrophages in early stages of breast tumor development in obese patients. In this study, the effects of pro-inflammatory macrophages on breast cancer–adipocyte crosstalk were investigated. Methods: An innovative human cell co-culture system was built and used to model the paracrine interactions among adipocytes, macrophages, and breast cancer cells and how they facilitate tumor progression. The effects on cancer cells were examined using cell counts and migration assays. Quantitative reverse-transcription polymerase chain reaction was used to measure the expression levels of several cytokines and proteases to analyze adipocyte cancer association. Results: Macrophage-conditioned media intensified the effects of breast cancer–adipocyte crosstalk. Adipocytes became delipidated and increased production of pro-inflammatory cytokines, even in the absence of cancer cells, although the expression levels were highest with all three cell components. As a result, co-cultured breast cancer cells became more aggressive, with increased proliferation and migration compared to adipocyte–breast cancer co-cultures treated with unconditioned media. Conclusions: A novel co-culture model was built to evaluate the crosstalk among human macrophages, adipocytes, and breast cancer cells. We found that macrophages may contribute to adipocyte inflammation and cancer association and thus promote breast cancer progression.

## 1. Introduction

Obesity is a growing problem affecting an estimated 650 million adults worldwide [1]. In the United States, 41.9% of adults were classified as obese in 2020 [2]. Obesity increases the risk of several types of cancer, including post-menopausal breast cancer [3]. Overweight and obese women were found to have 1.5 and 2 times greater relative risks of post-menopausal breast cancer, respectively. Obesity is also a poor prognosis marker for several cancers and causes increased death rates [4,5,6]. It is estimated that 14% of cancer deaths in men and 20% in women are related to obesity [5].

Breast cancer is the second most commonly diagnosed cancer and the second leading cause of cancer deaths for women in the United States. While the majority of research focuses on breast cancer cells, now it is understood that the tumor microenvironment plays an important role in cancer progression and prognosis. In breast cancer tissue, tumor cells are in close proximity to adipocytes. Adipocytes in contact with tumor cells show distinctive phenotypic changes and altered secretion profiles, which have been termed cancer-associated adipocytes (CAAs) [7]. CAAs undergo reductions in size and lipid content and upregulate expression of pro-inflammatory cytokines, such as interleukin-6 (IL-6), interleukin-1β (IL-1β), and tumor necrosis factor-α (TNF-α) [7]. Proteases that can remodel the extracellular matrix (ECM), such as matrix metalloproteinase-11 (MMP-11), are also upregulated [7]. MMPs can aid in cancer invasion and metastasis. CAAs in turn support tumor growth, migration, and invasion [7,8,9], resulting in a process known as breast cancer–adipocyte crosstalk. This may also help explain the worse prognosis in obese breast cancer patients.

In addition to more adipocytes, obesity also results in increased infiltration of macrophages into adipose tissue [10,11,12]. Adipocytes secrete monocyte chemoattractant protein-1 (MCP-1), which leads to macrophage infiltration, and other pro-inflammatory cytokines that establish a state of chronic low-grade inflammation. Macrophages recruited into adipose tissue are pro-inflammatory, secreting cytokines such as IL-1β and TNF-α, and contribute to the chronic inflammation seen in obese people [12]. Many studies have demonstrated that established tumors can cause macrophages to undergo anti-inflammatory supportive polarization [13,14]. These anti-inflammatory macrophages, referred to as M2 macrophages, then benefit tumor growth and survival by shielding cancer from the immune system [13,14]. However, little research has focused on the effects of macrophages in the early stages of tumor development in obese patients. In this study, we investigated the effects of pro-inflammatory macrophages on breast cancer–adipocyte crosstalk.

## 2. Materials and Methods

### 2.1. Cell Culture

Adult human mesenchymal stem cells (hMSCs) were obtained from the Tulane Center for Gene Therapy. Human MSCs were grown in Minimum Essential Medium, Eagle Alpha Modification (α-MEM; Sigma, St. Loius, MO, USA; M0644), supplemented with 20% fetal bovine serum (FBS; Seradigm, Radnor, PA, USA) and 2.2 g/L sodium bicarbonate. An L-glutamine, penicillin, and streptomycin cocktail (Sigma; G1146) was also added for a final concentration of 200 mM L-glutamine, 10,000 units/mL penicillin, and 100 mg/mL streptomycin. The human U937 cell line was obtained from the American Type Culture Collection (ATCC, Manassas, VA, USA; CRL-1593.2) and grown in RPMI-1640 media (Sigma; R8755) supplemented with 10% FBS, 2.0 g/L sodium bicarbonate, and the L-glutamine, penicillin, and streptomycin cocktail. MCF-7 human breast cancer cells were obtained from the ATCC (HTB-22) and grown in α-MEM supplemented with 10% FBS, 2.2 g/L sodium bicarbonate, and the L-glutamine, penicillin, and streptomycin cocktail. MDA-MB-231 human breast cancer cells were obtained from the ATCC (HTB-26) and grown in α-MEM supplemented with 10% FBS, 2.2 g/L sodium bicarbonate, and the L-glutamine, penicillin, and streptomycin cocktail. Cells were maintained in a fully humidified incubator at 37 °C with 5% CO_2_.

### 2.2. Differentiation of hMSCs into Adipocytes

Human MSCs were plated at 1000 cells/cm^2^ in 6-well plates for 2D cell culture and 2000 cells per well in 96-well plates for 3D spheroid culture. Monolayer cultures were maintained in the α-MEM media described above until 70% confluency was reached. Then, adipocyte differentiation was induced through the addition of 0.5 µM dexamethasone (Sigma; D4902), 0.5 µM isobutylmethylxanthine (Sigma; I7018), and 50 µM indomethacin (Sigma; I7378) to the culture media [15,16]. After 21 days of differentiation, the cells were used for further experimentation.

### 2.3. Differentiation of U937 Cells into Macrophages

U937 cells were induced towards a macrophage phenotype via the addition of phorbol myristate acetate (PMA; BioVision, San Francisco, CA, USA; 1544-5) to the media [17,18]. Various concentrations (0–100 ng/mL) and timepoints (24 or 48 h) were examined to determine optimal differentiation (Figure S1). Following treatment, cells were cultured for an additional 72 h in untreated media. Differentiation was determined by assessing the morphology and attachment of the cells. Non-adherent cells were removed, and adherent cells were counted using a Cedex HiRes cell analysis system (Innovatis Inc., Fairfax, VA, USA). A quantity of 100 ng/mL PMA for 48 h was determined to yield the most differentiated cells, which were subsequently used for all experiments.

### 2.4. Macrophage-Conditioned Media (CM) Preparation

Once differentiation conditions were optimized, 1 million U937 cells/mL were plated in 10 cm dishes. Then, 100 ng/mL PMA was added. After 48 h, PMA was removed and cells were cultured in untreated media for 72 h. Then, media was changed and conditioned for 24 h. Media was then collected and filtered through a 0.2-micron filter. Conditioned media (CM) was stored at −20 °C until use.

### 2.5. Breast Cancer–Adipocyte Co-Culture

**Figure 1:**
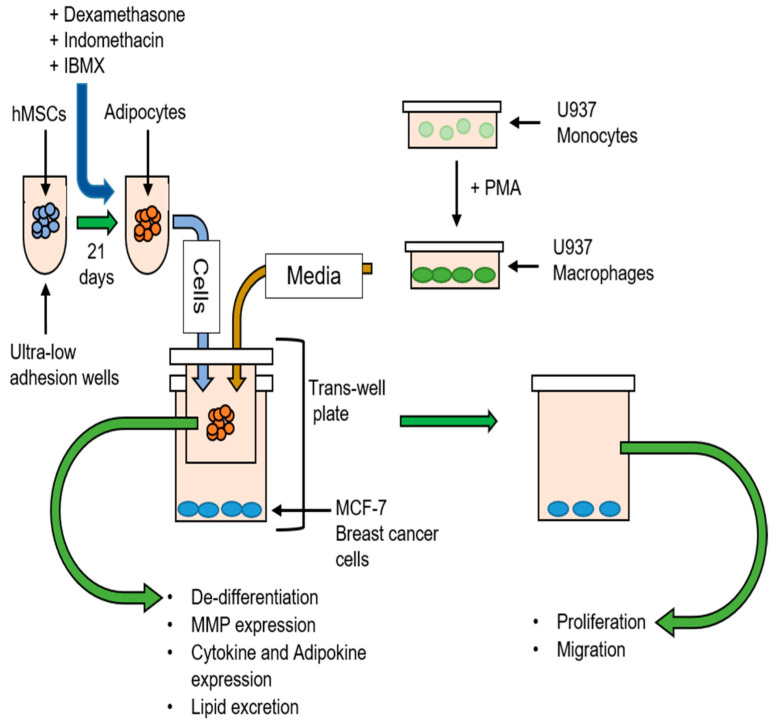
Human cell co-culture scheme. hMSCs were used to make human adipocytes and placed in co-culture with MCF-7 breast cancer cells. Monocytes were used to make macrophages, and conditioned media was used in co-culture. Breast cancer cells were tested for proliferation and migration, while adipocytes were tested for lipid loss and cytokine and MMP expression.

MCF-7 breast cancer cells were co-cultured with adipocytes in macrophage-conditioned media (Figure 1). Twenty-four-well dishes were used for breast cancer cell proliferation and migration assays. Six-well dishes were used for RT-PCR experiments. Transwells of corresponding sizes were used to keep the cell types apart. MCF-7 cells were plated at 2 × 10^4^ cells/cm^2^. Adipocytes were plated at 2 × 10^4^ cells in 24-well dishes, which corresponded to 10 spheres of 2000 cells/sphere. For qRT-PCR, adipocytes were plated in normal 2D culture, as described above. Macrophage-conditioned media was used 1:1 with regular growth media. MCF-7 cells co-cultured with adipocytes in unconditioned media served as controls. MCF-7 cells were also grown alone in macrophage-conditioned media as an additional control. After 3 days, the system was taken apart, and each cell type was examined individually. All co-cultures were left together for 3 days unless otherwise specified. The same methods were used for co-culture experiments using the MDA-MB-231 cell line.

### 2.6. Breast Cancer Cell Counts

All cell counts were made using a Cedex HiRes cell analysis system. Trypan blue was used for live/dead staining. All experiments were run in triplicate. To determine viability, live cell counts were divided by total cell counts for each condition.

### 2.7. Wound Healing Assay

After 3 days in co-culture, the adipocytes were removed and a scratch in the shape of a cross was made on the confluent breast cancer cell layer using a 200 µL pipette tip. The cells were washed with PBS, and fresh media was added. Pictures were taken, centered on the center of the cross for reference, and the cells were left to recover for 24 h, after which pictures were taken again. The area was quantified using the MRI wound healing tool in ImageJ and represented as percentage of wound closed after 24 h. All experiments were run in triplicate.

### 2.8. Real-Time Quantitative Reverse-Transcription Polymerase Chain Reaction (qRT-PCR)

After PMA treatment, macrophage cells were left to recover for 48 h and then harvested for qRT-PCR. RNA was isolated from the cells using the E.Z.N.A. Total RNA Kit I (Omega Bio-Tek, Norcross, GA, USA; R6834) and purified with the RNA Clean & Concentrator Kit (Zymo Research, Irvine, CA, USA; R1013). A quantity of 1 ug of RNA was converted to cDNA using the qScript cDNA SuperMix (Quanta Bioscience, Beverly, MA, USA; 95048). A quantity of 100 ng/μL cDNA was used as a template for quantitative PCR on a 7500 Fast Real Time PCR system (Applied Biosystems, Foster City, CA, USA), using SYBR green PCR master mix (Applied Biosystems; 4344463). The PCR was run as follows: one cycle of 2 min at 50 °C and 10 min at 95 °C, then 40 cycles of 15 s at 95 °C, 30 s at 55 °C, and 30 s at 68 °C. Relative gene expression was determined using the 2^(−ΔΔCt)^ method [19]. Primers were ordered from Eurofins (Luxembourg), and GAPDH was used as an endogenous control. Primer sequences are shown in Table 1.

For adipocytes, 2D cultures were placed either in co-culture with MCF-7 cells or alone and either in macrophage-conditioned media or non-conditioned media. The adipocytes were harvested after 6 and 24 h, and the samples were prepped for qRT-PCR by the same method described above.

### 2.9. Statistical Analysis

Experiments were performed with at least three independent samples per data point. Data analysis was performed in Excel using Student’s *t*-tests. Proliferation, viability, migration, differentiation, and qRT-PCR results are expressed as means ± standard errors.

## 3. Results

### 3.1. Quantitative RT-PCR Analysis of U937 Monocyte-Derived Macrophages

**Figure 2:**
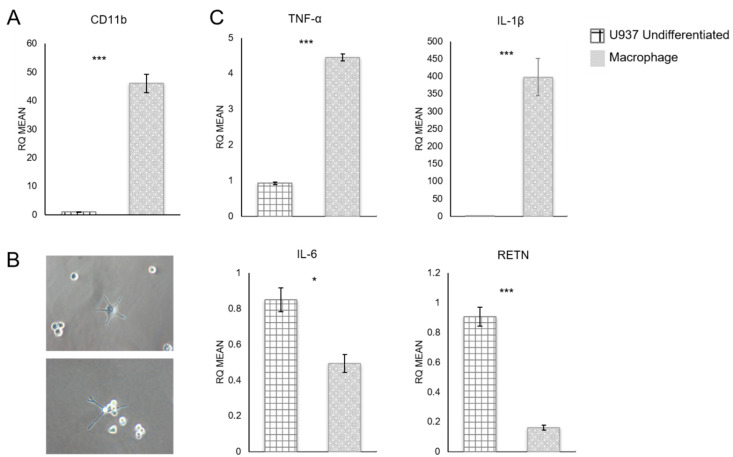
U937 cell gene expression levels measured by qRT-PCR. (**A**) Expression levels of CD11b in undifferentiated and macrophage-differentiated U937 cells. CD11b was significantly upregulated after differentiation. (**B**) Differentiated cells showed morphological changes, from suspension cells to attached, macrophage-like cells. Magnification 200×. (**C**) TNF-α and IL-1β expression increased after differentiation, while IL-6 and RETN expression decreased. RQ = relative quantification. * *p* ≤ 0.05, *** *p* ≤ 0.001. Data presented as means ± SEMs.

Differentiated U937 cells were analyzed for expression of macrophage marker CD11b [17], which was greatly increased after PMA treatment (*p*-value = 0.0004) (Figure 2A). Differentiated cells also showed changes in morphology (Figure 2B) consistent with a macrophage phenotype. More specifically, after differentiation, U937 cells became adherent and displayed pseudopodia.

Expression levels of several cytokines of interest were quantified using qRT-PCR (Figure 2C). Differentiated macrophages showed significantly increased levels of TNF-α (*p*-value = 0.0006) and IL-1β (*p*-value = 0.004) and significantly decreased expression levels of IL-6 (*p*-value = 0.025) and RETN (*p*-value = 0.0007) (Figure 2C). However, although IL-6 and RETN expression decreased, these pro-inflammatory cytokines were still produced by the macrophages. Therefore, these macrophages secrete pro-inflammatory cytokines like the classical M1 pro-inflammatory macrophages that are present in breast tissue.

### 3.2. Breast Cancer Cell Proliferation and Migration

**Figure 3:**
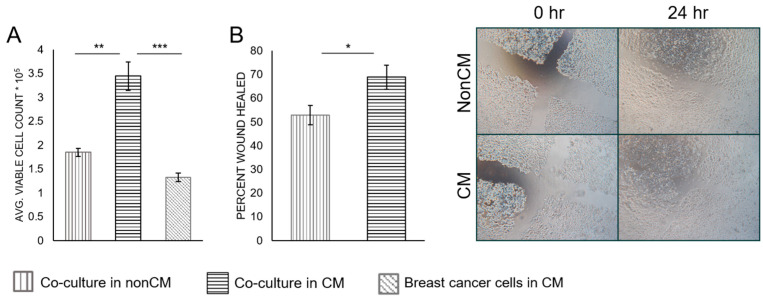
MCF-7 proliferation (**A**) and wound healing assay (**B**). Cell counts were used to determine proliferation after 3 days in co-culture with adipocytes, with macrophage-CM or without macrophage-CM, or in CM alone. After 3 days in co-culture with adipocytes, with macrophage-CM or without macrophage-CM, a scratch was made on the MCF-7 confluent cell layer and migration was measured after 24 h. * *p* ≤ 0.05, ** *p* ≤ 0.01, *** *p* ≤ 0.001. Data presented as means ± SEMs.

Using the co-culture model described in Figure 1, the effects of macrophage-CM on breast cancer cells were examined. MCF-7 cells in co-culture with adipocytes in macrophage-CM showed a significant increase in cell number after 3 days when compared with both cells grown in CM alone (0.001) and in co-culture with nonconditioned media (*p*-value = 0.004) (Figure 3A). Cell viability was similar between all conditions (Figure S2). MCF-7 cells co-cultured with adipocytes in macrophage-CM also showed increased migration capabilities when compared with cells co-cultured in non-CM (*p*-value = 0.02) (Figure 3B).

The effects of adipocyte co-culture and macrophage-CM were then investigated with the more aggressive, triple-negative breast cancer cell line MDA-MB-231 for comparison (Figure S3). Unlike the MCF-7 cells, MDA-MB-231 cells did not show a significant increase in cell proliferation (*p*-value = 0.098) or migration (*p*-value = 0.4) when macrophage-CM was added to the co-culture system (Figure S3). Viability was again similar across all conditions (Figure S4). However, MDA-MB-231 cells did show increased proliferation when in co-culture with adipocytes compared to macrophage-CM (*p*-value = 0.03) (Figure S3).

### 3.3. Quantitative RT-PCR Analysis of Adipocytes

**Figure 4:**
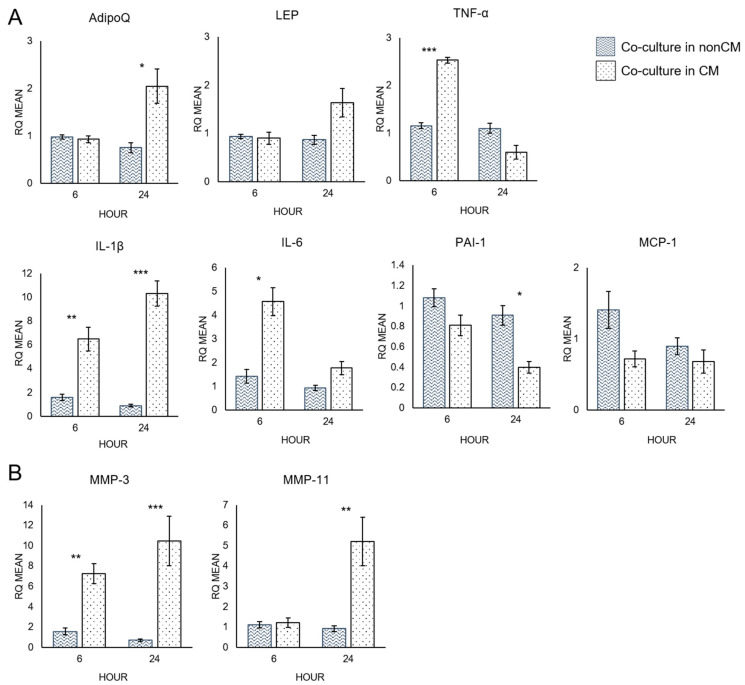
Cytokine (**A**) and MMP (**B**) mRNA expression of adipocytes in co-culture with breast cancer cells with macrophage-CM or without macrophage-CM, at 6 and 24 h. RQ = relative quantification. * *p* ≤ 0.05, ** *p* ≤ 0.01, *** *p* ≤ 0.001. Data presented as means ± SEMs.

Quantitative RT-PCR analysis was used to measure the mRNA expression levels of several cytokines and proteases of interest that indicate delipidation of adipocytes (Figure 4). Adiponectin (AdipoQ), which is inversely correlated with fat mass, was significantly increased after 24 h in co-culture with MCF-7 BRCA cells with macrophage-CM compared to co-culture in non-CM (*p*-value = 0.02) (Figure 4A). This indicated a decrease in the lipid contents of the adipocytes when macrophage-CM was added to the co-culture. Plasminogen activator inhibitor-1 (PAI-1), which is directly correlated with fat mass, decreased significantly after 24 h (*p*-value = 0.018). All the measured pro-inflammatory cytokines (IL-1β, IL-6, and TNF-α) increased when macrophage-CM was added to the co-culture system. IL-6 (*p*-value = 0.014) and TNF-α (*p*-value = 0.0005) gene expression spiked early, after 6 h, while IL-1β (*p*-value = 0.0003) continued to increase over 24 h. TNF-α can cause delipidation of adipocytes [20], and levels increased early, before the rise in AdipoQ, which suggests decreased lipid contents in the co-culture exposed to macrophage-CM. qRT-PCR analysis was also used to test for MMP-3 and MMP-11 levels (Figure 4B). MMP-3 and MMP-11 are both negative regulators of adipogenesis, and MMP-11 can cause delipidation of adipocytes [21,22]. MMP-3 expression levels increased significantly after both 6 (*p*-value = 0.007) and 24 h (*p*-value = 0.002). MMP-11 also increased after 24 h (*p*-value = 0.007).

**Figure 5:**
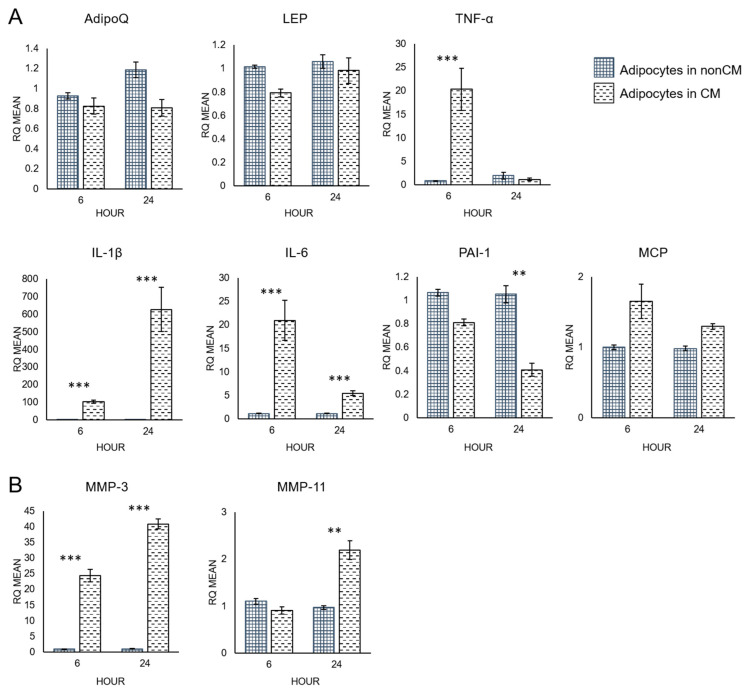
Cytokine (**A**) and MMP (**B**) expression of adipocytes not in co-culture with macrophage-CM or without macrophage-CM, at 6 and 24 h. RQ = relative quantification. ** *p* ≤ 0.01, *** *p* ≤ 0.001. Data presented as means ± SEMs.

Additionally, adipocytes cultured alone with or without macrophage-CM were analyzed by qRT-PCR to study the direct effects of macrophage-CM on adipocytes (Figure 5). AdipoQ and leptin (LEP) mRNA expression levels were similar across both conditions (Figure 5A). However, expression of pro-inflammatory cytokines, IL-6, TNF-α, and IL-1β, increased in response to macrophage-CM. IL-1β showed the largest difference after 24 h (*p*-value = 0.00001), while IL-6 (*p*-value = 0.0007) and TNF-α (*p*-value = 0.0004) peaked at 6 h. PAI-1 expression decreased significantly after 24 h (*p*-value = 0.006). Monocyte chemoattractant protein-1 (MCP-1) expression was not altered.

Macrophage-CM also increased expression of MMPs in adipocytes cultured without breast cancer cells (Figure 5B). MMP-3 was significantly higher after 6 h (*p*-value = 0.00001) and continued to increase over 24 h (*p*-value = 0.0000004). MMP-11 expression was significantly increased after 24 h (*p*-value = 0.003).

## 4. Discussion

Obesity causes systemic low-grade inflammation, which in turn increases cancer incidence and mortality. In breast tissue, obesity also results in increased infiltration of macrophages that contribute to inflammation. Many studies have shown that cancer cells can cause delipidation and cancer association of adipocytes in the breast cancer microenvironment [7,23]. The ability of established tumors to stimulate M2 conversion in macrophages is also well-documented [24]. However, the effects of nascent pro-inflammatory M1 macrophages on breast cancer–adipocyte crosstalk in early tumorigenesis is largely unknown.

### 4.1. Macrophage-Conditioned Media Has Varying Effectiveness upon Different Types of Breast Cancer Cells

To more accurately model the human breast cancer microenvironment, we developed a novel co-culture system that utilized only human-derived cells and could model the breast cancer–adipocyte crosstalk in an obese and a lean patient. Our model could also examine the contribution of infiltrating macrophages. The luminal A cell line MCF-7 was principally used, since it is hormone-receptor-positive and not metastatic. This non-aggressive cell line offers a better representation of early tumorigenesis, which is usually less aggressive than in established tumors. MCF-7 cells grown in co-culture with macrophage-CM showed higher proliferation and migration compared to breast cancer cells in co-culture without macrophage-CM (Figure 3). MCF-7 cells were also cultured alone in macrophage-CM, which did not cause increased cell proliferation (Figure 3A). Therefore, this effect was not due solely to the pro-inflammatory cytokines secreted by the macrophages, such as IL-1β and TNF-α (Figure 2C), which can benefit tumor cell proliferation. Instead, the macrophage-secreted factors affected the adipocytes, which in turn affected the breast cancer cells. MCF-7 cells grown in co-culture without macrophage-CM also showed higher proliferation than the cancer cells alone, which agrees with previous studies that showed increased tumor growth as a result of breast cancer–adipocyte crosstalk [7,9]. However, our study shows that this effect is even stronger when macrophage-CM media is added. This indicates that macrophages that infiltrate breast adipose tissue could contribute to early tumorigenesis, before the tumor cells stimulate M1-to-M2 conversion.

This study’s main focus was on the effect of M1 macrophage infiltration in priming an inflammatory microenvironment in early tumorigenesis. However, to see if this would also affect a more aggressive, established tumor, the breast cancer proliferation and migration studies were repeated using the MDA-MB-231 cell line (Figure S3). MDA-MB-231 is a triple-negative, highly aggressive, and metastatic breast cancer cell line. In co-culture, adipocytes again promoted the growth of the cancer cells. However, in contrast to MCF-7 cells, macrophage-CM did not appreciably increase MDA-MB-231 cell growth or migration. One possible explanation for the observed difference is the estrogen receptor status of these cell lines. MCF-7 cells are estrogen-receptor-positive, while MDA-MB-231 cells are estrogen-receptor-negative. Adipocytes synthesize estrogen through aromatase-mediated metabolism of androgen precursors in post-menopausal women [25]. Macrophage-derived proinflammatory mediators can also induce aromatase- and estrogen-dependent gene expression in adipocytes [25]. Therefore, estrogen overexpression by adipocytes could be one mechanism by which cancer-associated adipocytes contribute to breast cancer progression.

### 4.2. Macrophage-Conditioned Media Increases the Pro-Inflammatory Character of Adipocytes

The expression levels of several cytokines associated with inflammation and breast cancer were highly up-regulated by adipocytes, both in co-culture and alone, in response to macrophage-CM. For instance, there were marked increases in the expression of the pro-inflammatory cytokines, IL-1β, TNF-α, and IL-6. This is consistent with another study by Permana et al., which also showed that murine RAW 264.7 macrophage-conditioned media could induce 3T3-L1 adipocyte inflammation [26,27]. The co-culture system used in our study consisted of only human cell lines, establishing that these effects are present in human cell interactions and can have important implications for human breast cancer progression and prognosis.

Curiously, however, increased expression of adiponectin, an anti-inflammatory cytokine that is secreted solely by mature adipocytes and is inversely correlated with fat mass, seemed to be more dependent upon co-culture with breast cancer cells than upon the addition of macrophage-CM. Increased adiponectin is an indicator of delipidation, which is one of the hallmarks of adipocyte cancer association [7,25]. TNF-α has also been reported to cause delipidation of adipocytes and may promote the expression of adiponectin [28,29]. However, when adipocytes were cultured alone in macrophage-CM, there was no significant increase in adiponectin, unlike in co-culture, even though TNF-α was increased. Consequently, it would seem that additional breast-cancer-cell-related factors play a substantial role in adiponectin expression and that all three cell types work in concert to cause adipocyte cancer association and promote tumor aggressiveness. One limitation of this work is that only mRNA levels were investigated. In future work, these changes in cytokine expression should be verified at the protein level as well.

### 4.3. Macrophage-Conditioned Media Increases Matrix Metalloproteinase Expression in Adipocytes

Macrophage-CM also increased MMP-3 and MMP-11 expression by adipocytes in adipocyte–breast cancer co-culture (Figure 4B). MMP-11 is a negative prognosis marker in several human cancers [30] and can also cause delipidation of adipocytes [20]. Andarawewa et al. found that MMP-11 was expressed in human adipocytes proximally located to invasive cancer cells but not in distally located adipocytes [20]. In addition, this same study found that these adipocytes at the tumor invasive front that expressed MMP-11 were reduced in size. In studies examining the role of MMPs in normal adipogenesis, MMP-11- and MMP-3-deficient mice developed more adipose tissue, indicating a negative regulatory role [21,22]. The increased expression of MMP-11 and MMP-3 further indicates that macrophage-CM can increase adipocyte delipidation and cancer association. Finally, while MMP-11 expression by adipocytes, both in co-culture and alone, was similarly affected by macrophage-CM, there appeared to be an additive effect on MMP-3 expression. Although MMP-3 and MMP-11 are structurally and functionally similar, both being stromelysins and negative regulators of adipogenesis, their expression may require different signaling mechanisms.

## 5. Conclusions

In conclusion, our results suggest that macrophage-derived factors can exacerbate the effects of human breast cancer cell–adipocyte crosstalk. Macrophage-CM resulted in increased breast cancer cell proliferation and migration and adipocyte cancer association. The influx of macrophages into adipose tissue in obese patients contributes to the inflammation caused by adipocytes and may prime the microenvironment for tumorigenesis. When breast cancer cells are added into this system, adipocytes produce even more pro-inflammatory cytokines and show signs of delipidation and promote tumor cell aggressiveness. These results are of particular importance for obese breast cancer patients, who have increased macrophage infiltration into adipose tissue. The effects of macrophages on this crosstalk may help account for the worse prognosis in obese breast cancer patients. Additionally, these findings indicate potential new treatments or drug targets for obese breast cancer patients. Limiting macrophage infiltration or reducing the secretion of macrophage proinflammatory cytokines could help reduce inflammation in breast tissue and make the microenvironment less favorable for tumorigenesis.

## Figures and Tables

**Table 1:** Primers used for qRT-PCR.

Gene	Forward Primer	Reverse Primer
CD 11b	5′-CTTCCAGGTTCTGGCTCCTTC-3′	5′-TCTCTTGGAAGGTCATTGCGTT-3′
MCP-1	5′-TCTCAAACTGAAGCTCGCACT-3′	5′-GGGAATGAAGGTGGCTGCTA-3′
IL-6	5′-CCTGCAGAAAAAGGCAAAGAATC-3′	5′-GAGTTGTCATGTCCTGCAGCC-3′
RETN	5′-TGCAGGATGAAAGCTCTCTGTCTC-3′	5′-CCTGGATCCTCTCATTGATGGC-3′
TNF-α	5′-TGGGATCATTGCCCTGTGAG-3′	5′-GGTGTCTGAAGGAGGGGGTA-3′
IL-1β	5′-CAGGCTGCTCTGGGATTCTC-3′	5′-AAGTCATCCTCATTGCCACTGT-3′
AdipoQ	5′-ATGGCCCCTGCACTACTCTA-3′	5′-CAGGGATGAGTTCGGCACTT-3′
LEP	5′-CCCTGGAGTGCAGTTTCCAA-3′	5′-TGCTCAGATGAACCCAACCC-3′
PAI-1	5′-TTGCAGGATGGAACTACGGG-3′	5′-GTGGCAGGCAGTACAAGAGTGA-3′
MMP-3	5′-CCATCTCTTCCTTCAGGCGT-3′	5′-ATGCCTCTTGGGTATCCAGC-3′
MMP-11	5′-ATGAATTTGGCCACGTGCTG-3′	5′-CGAAAGGTGTAGAAGGCGGA-3′

## Data Availability

The datasets generated and analyzed in the current study are available in this publication and in the Supplementary Materials.

## Supplementary Materials

The following supporting information can be downloaded at: https://www.mdpi.com/article/10.3390/biom12121757/s1, Figure S1: Number of adherent cells after PMA treatment. U937 monocytes were treated with 0–100 ng/mL PMA and left for 24 or 48 h. Macrophage differentiation was determined by cell adherence. 100 ng/mL PMA for 48 h was determined to yield the highest differentiation rate and was used for subsequent experiments. Error bars denote standard error; Figure S2: MCF-7 average cell viability remained unchanged for cell count assays. Error bars denote standard error; Figure S3: MDA-MB-231 cell proliferation and migration. Cell counts were used to determine proliferation after 3 days in co-culture with adipocytes, with macrophage CM or without macrophage CM, or alone in CM. After 3 days in co-culture with adipocytes, with macrophage CM or without macrophage CM, a scratch was made on the confluent MDA-MB-231 cell layer and migration was measured after 24 h. Asterisk denotes significant p-value. * means *p*-value ≤ 0.05, ** means *p*-value ≤ 0.01, and *** means *p*-value ≤ 0.001. Error bars denote standard error; Figure S4: MDA-MB-231 average viability remained unchanged for cell count assays. Error bars denote standard error.

## Abbreviations

α-MEM	Minimum Essential Medium: Eagle Alpha Modification
AdipoQ	Adiponectin
BRCA	Breast cancer
CAA	Cancer-associated adipocytes
CD11b	Cluster of differentiation molecule 11B
CM	Conditioned media
ECM	Extracellular matrix
FBS	Fetal bovine serum
GAPDH	Glyceraldehyde 3-phosphate dehydrogenase
HMSCs	Human mesenchymal stem cells
IL-1β	Interleukin-1β
IL-6	Interleukin-6
LEP	Leptin
MMP-3	Matrix metalloproteinase-3
MMP-11	Matrix metalloproteinase-11
NonCM	Non-conditioned media
PAI-1	Plasminogen activator inhibitor-1
PMA	Phorbol myristate acetate
RETN	Resistin
RQ	Relative quantification
TNF-α	Tumor necrosis factor-α
