# Supplementary material for: Macrophage-Conditioned Media Promotes Adipocyte Cancer Association, Which in Turn Stimulates Breast Cancer Proliferation and Migration
Source: Biomolecules. 2022 Nov 26;12(12):1757. doi: 10.3390/biom12121757 (PMC9775594; doi:10.3390/biom12121757)
Supplement: Supplementary file 1 [file biomolecules-12-01757-s001.zip › biomolecules-2010934-supplementary.pdf]

Supplementary Information

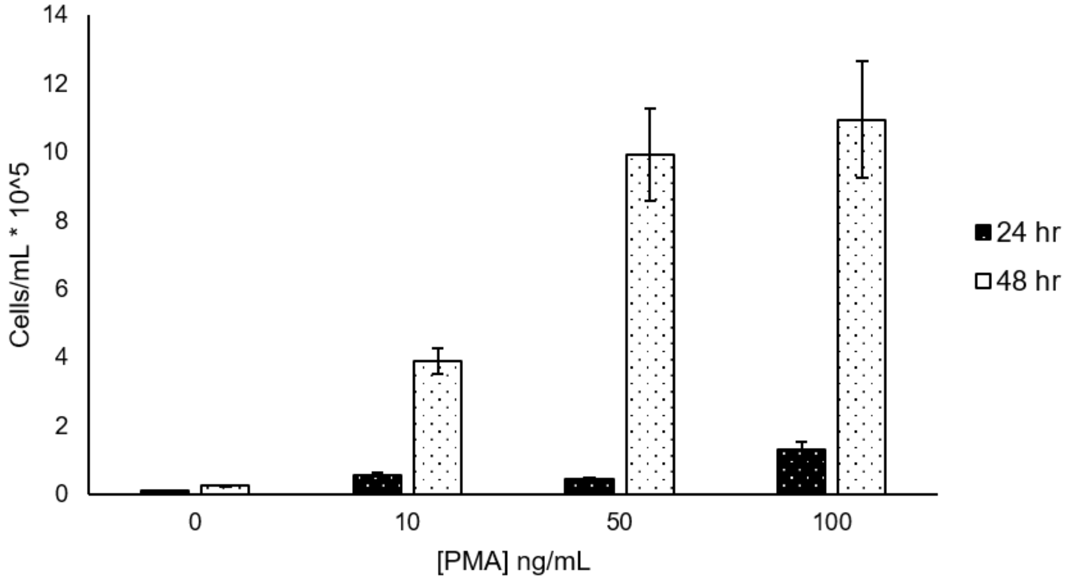

Figure S1: Number of adherent cells after PMA treatment. U937 monocytes were treated with 0-100 ng/mL PMA and left for 24 or 48 hours. Macrophage differentiation was determined by cell adherence. 100 ng/mL PMA for 48 hours was determined to yield the highest differentiation rate and was used for subsequent experiments. Error bars denote standard error.

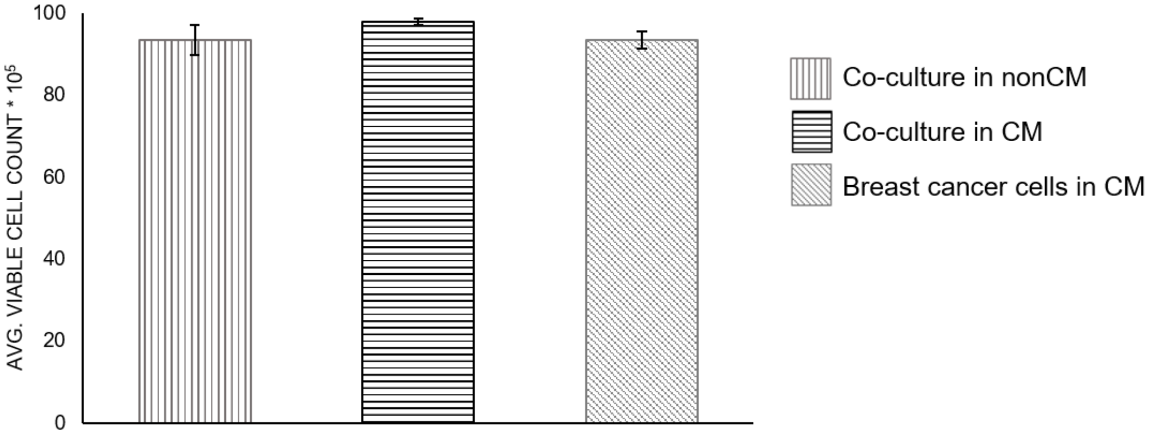

Figure S2: MCF-7 average cell viability remained unchanged for cell count assays. Error bars denote standard error.

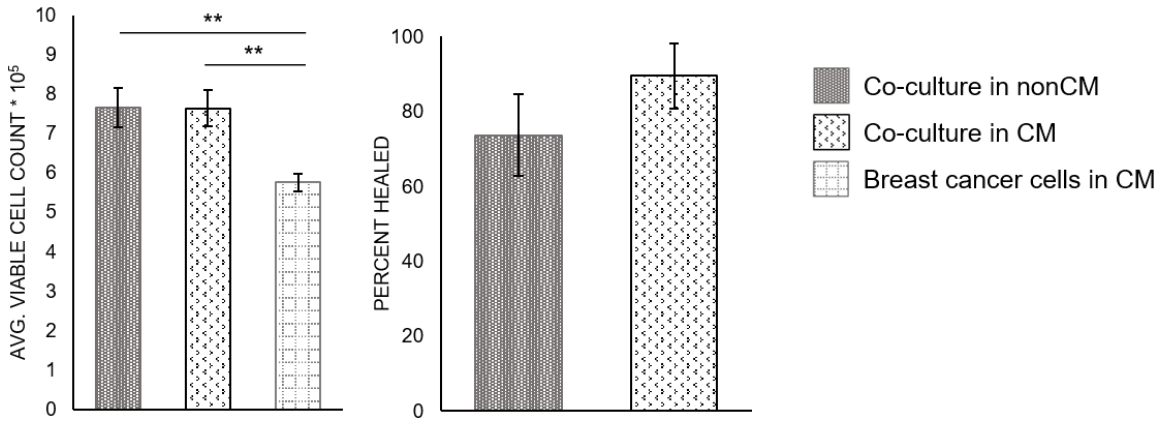

Figure S3: MDA-MB-231 cell proliferation and migration. Cell counts were used to determine proliferation after 3 days in co-culture with adipocytes, with macrophage CM or without macrophage CM, or alone in CM. After 3 days in co-culture with adipocytes, with macrophage CM or without macrophage CM, a scratch was made on the confluent MDA-MB-231 cell layer and migration was measured after 24 hours. Asterisk denotes significant p-value. ** means p-value ≤ 0.01. Error bars denote standard error.

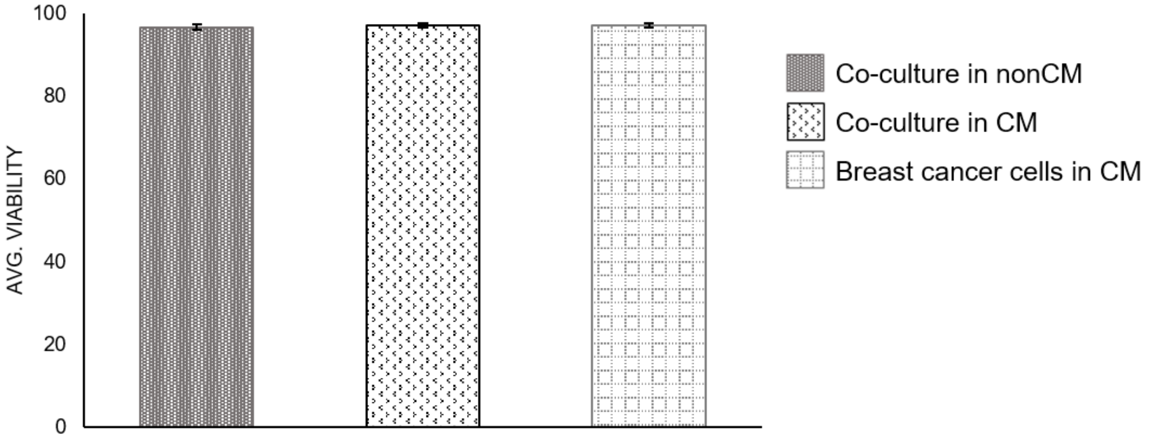

Figure S4: MDA-MB-231 average viability remained unchanged for cell count assays. Error bars denote standard error.
